# Yeast-based reference materials for quantitative metabolomics

**DOI:** 10.1007/s00216-021-03694-w

**Published:** 2021-10-13

**Authors:** Hendri Wasito, Gerrit Hermann, Veronika Fitz, Christina Troyer, Stephan Hann, Gunda Koellensperger

**Affiliations:** 1 Institute of Analytical Chemistry, Department of Chemistry, University of Natural Resources and Life Sciences (BOKU) Vienna, Muthgasse 18, 1190 Vienna, Austria; 2 Department of Pharmacy, Faculty of Health Sciences, Jenderal Soedirman University, Dr. Soeparno Street, 53122 Purwokerto, Indonesia; 3 Core Facility Mass Spectrometry, University of Natural Resources and Life Sciences (BOKU) Vienna, Muthgasse 11, 1190 Vienna, Austria; 4 ISOtopic Solutions, Waehringer Str. 38, 1090 Vienna, Austria; 5 Department of Analytical Chemistry, Faculty of Chemistry, University of Vienna, Waehringer Str. 38, 1090 Vienna, Austria; 6 Vienna Metabolomics Center (VIME), University of Vienna, Althanstraße 14, 1090 Vienna, Austria

**Keywords:** Metabolomics, Reference material, *Pichia pastoris*, Targeted analysis, Harmonization, Absolute quantification

## Abstract

We introduce a new concept of yeast-derived biological matrix reference material for metabolomics research relying on in vivo synthesis of a defined biomass, standardized extraction followed by absolute quantification with isotope dilution. The yeast *Pichia pastoris* was grown using full control- and online monitoring fed-batch fermentations followed by fast cold methanol quenching and boiling ethanol extraction. Dried extracts served for the quantification campaign. A metabolite panel of the evolutionarily conserved primary metabolome (amino acids, nucleotides, organic acids, and metabolites of the central carbon metabolism) was absolutely quantified by isotope dilution utilizing uniformly labeled ^13^C-yeast-based internal standards. The study involved two independent laboratories employing complementary mass spectrometry platforms, namely hydrophilic interaction liquid chromatography-high resolution mass spectrometry (HILIC-HRMS) and gas chromatography-tandem mass spectrometry (GC–MS/MS). Homogeneity, stability tests (on a panel of >70 metabolites over a period of 6 months), and excellent biological repeatability of independent fermentations over a period of 2 years showed the feasibility of producing biological reference materials on demand. The obtained control ranges proved to be fit for purpose as they were either superior or comparable to the established reference materials in the field.

**Supplementary Information:**

The online version contains supplementary material available at 10.1007/s00216-021-03694-w.

## Introduction

The emergence of large-scale metabolomics screenings in clinical research and other regulated environments calls for harmonization. Standardized methods are key as emphasized by massive joint efforts of international societies (e.g., the Metabolomics Standardization Initiative (MSI) of the Metabolomics Society [1]) deploying community guidelines [1–6]. Definitions for minimum quality requirements together with harmonized protocols for measurement and reporting are established. Quantitative measurements require validation schemes integrating standards and reference materials. In -omics type of analysis, this concept is gaining significant momentum; however, the pace of developing routine applications is slow due to the lack and the costs of standards [7]. For large-scale metabolomics and lipidomics studies, the use of pooled samples is proposed as a minimum requirement improving intra- and inter-batch repeatability [8, 9]. The integration of multi-standard panels in ready to use, well-plate formats proofed to be a valuable strategy [7]. Moreover, interlaboratory comparisons and the wide adoption of kit-type analysis advanced harmonization. Guidelines and protocols are key steps towards harmonization; however, inter-assay commutability and ultimately traceability are only achieved by integrating (certified) reference materials. To date, metabolomics methods of the highest metrological order are restricted to the few existing traceable certified reference material in the field [7]. The first multi-metabolite standard pushing in this direction was the standard reference material (SRM) 1950 developed by the National Institute of Standards and Technology (NIST, U.S. Department of Commerce: Gaithersburg, MD). It provides certified concentration values for approximately 100 small-molecule metabolites and environmental contaminants in human plasma [10]. Few matrix-based reference materials provided by metrological institutions or by accredited material producers followed, namely urine, blood, serum, plasma, and yeast [11]. Several seminal studies “repurposed” the SRM 1950 material and expanded the number of metabolite/lipids providing consensus values by interlaboratory comparisons [12, 13] or indicative values as obtained by one method in a single laboratory [14, 15]. E.g., an international ring trial established consensus values for 250 metabolites (amino acids, biogenic amines, acylcarnitines, glycerolipids, glycerophospholipids, cholesteryl esters, sphingolipids, hexoses) using a commercial kit [16]. Evidently, the production of omics-type reference material is inherently complex, given the number of metabolites and their vastly different chemistries. Thus, extensive studies on stabilizing conditions, resulting stability, and homogeneity are a prerequisite for reporting quantitative values with their estimated uncertainty. Recently, a novel strategy in producing long-term reference materials was presented [17]. The developed reference material relied on independently grown *Escherichia coli* batches, which were pooled following an iterative protocol. The method coined as iterative batch averaging method (IBAT) produced a stable and sustainable RM over time.

In this work, we investigated an alternative route of producing biological reference materials. The idea is to exploit in vivo synthesis for generating reference materials on demand and reproducibly. More specifically, we explore a yeast-derived reference material obtained from fully controlled fed-batch fermentations [18, 19], with glucose as sole carbon source. The material was already successfully implemented as benchmarking strategy. The in vivo synthesized metabolite library enabled in-house routines for instrumental performance tests (in analogy to the *HeLa* cell extracts in proteomics) and served for framing the chemical space and coverage upon method development [19]. In this work, we develop the yeast-based reference material further by adding the dimension of absolute quantities. The presented study involved the analysis of several completely independent fermentation batches over 2 years by complementary MS platforms in two independent laboratories. We showcase that the production of biological reference materials on demand is feasible for a set of > 50 metabolites, by controlling the fermentation conditions and standardizing extraction. We would like to denote our idea as next-generation reference materials accounting for the fact that certification would involve not only the material itself but also the whole production of the material as proposed by the standard ISO 17034 [20].

## Material and methods

### Chemical

LC–MS grade water and acetonitrile were provided by Sigma-Aldrich (Vienna, Austria) or Fisher Scientific (Vienna, Austria). The eluent additive of LC–MS grade formic acid was ordered from VWR International (Vienna, Austria). Other additives for HILIC-HRMS, such as ammonium hydroxide, ammonium bicarbonate, and ammonium formate, were purchased from Sigma-Aldrich (Vienna, Austria). Ethoxyamine hydrochloride, pyridine, and N-methyl-N-(trimethylsilyl)trifluoroacetamide with 1% trimethylchlorosilane were purchased for GC–MS/MS derivatization from Sigma-Aldrich (Vienna, Austria) and MachereyNagel (Macherey–Nagel GmbH, Dueren, Germany). Metabolite standards were purchased from Sigma-Aldrich (Vienna, Austria), Merck (Darmstadt, Germany), or Carbosynth (Berkshire, UK). All standards were accurately weighed and dissolved in an appropriate solvent. A multi-metabolite standard mixture that contained 148 metabolites for HILIC-HRMS was prepared by reconstitution in LC–MS grade water. For GC–MS/MS measurement, we used a mixture of 47 metabolite standards. Finally, a fully ^13^C-labeled internal standard yeast extract (isotopic enrichment > 99%) provided by ISOtopic solutions e.U. (Vienna, Austria) was added to both standard mixtures before a final dilution to 1:10 (v/v) using 80% acetonitrile and for LC–MS grade water for GC–MS/MS measurements, respectively.

### Preparation of in-house reference material

The investigated reference material was produced in-house in collaboration with ISOtopic solution e.U. after an adapted protocol from Neubauer et al. [18]. Briefly, the yeast *P. pastoris* was cultivated in a New Brunswick BioFlo 310 fed-batch fermentor for 72 h (Eppendorf, Hamburg, Germany) with full control over the input variables in terms of glucose as carbon source (Sigma-Aldrich, Steinheim, Germany), pH, temperature, and oxygenation. Process monitoring was facilitated by online measurement of pH, temperature, and dissolved oxygen. Offline assessment of optical density at 600 nm (OD_600_) and optical cell counting was performed at several time points. At the end of the process, the biomass was quenched in 60% methanol (v/v) at − 30 °C and subsequently extracted in boiling 80% ethanol (v/v) for metabolites. Finally, the ethanolic extract was aliquoted and dried in a vacuum centrifuge (Scan Speed 40, Labogene). Each dried aliquot corresponded to approximately 2 × 10^9^ yeast cells (corresponding to 15 mg cell dry weight). For measurement, they were reconstituted in 2 mL LC–MS grade water and vortexed for 15 min.

### HILIC-HRMS and GC–MS/MS measurements

A dried aliquot of the in-house produced reference material and one vial fully ^13^C-labeled internal standard both derived from approximately 2 × 10^9^ yeast cells (corresponding to 15 mg cell dry weight) separately reconstituted in 2 mL LC–MS grade water and vortexed for 15 min. The analytical samples consisted of both yeast and ^13^C-enriched yeast. The mixture solutions were completely dissolved for LC–MS before further measurements by vortexing for 0.5 min, followed by 1:10 dilution using 80% acetonitrile. On the other hand, the analytical samples that consisted of undiluted in-house reference material and 1:10 dilution of fully ^13^C-labeled internal standard solutions were completely dried after the addition of ethoxyamine hydrochloride in pyridine for protection of carbonyl groups during evaporation step before further automated just in time online two-step derivatization for GC–MS/MS measurements. The quantification strategy involved external calibration of a standard mixture with the addition of fully ^13^C-labeled yeast extract as an internal standard for HILIC-HRMS and GC–MS/MS measurements.

Hydrophilic liquid chromatography (HILIC) coupled to high-resolution MS was performed to simultaneously quantify small-molecule metabolites using the modified method from Schwaiger et al. [15, 21]. Briefly, an Acquity UPLC BEH Amide column (2.1 × 100 mm, 1.7 μm, Waters, Milford, USA) was used with gradient elution at 40 °C. Mobile phase A was 50 mmol L^−1^ ammonium formate in water with pH 4.0, and mobile phase B was acetonitrile/water 4:1 (v/v) with 50 mmol L^−1^ ammonium formate with pH 4.0. A Vanquish Duo UHPLC system (Thermo Scientific, Waltham, USA) followed by a high field Q Exactive HF quadrupole Orbitrap HRMS (Thermo Fisher Scientific) equipped with an electrospray ion source was used at a flow rate of 0.250 mL min^−1^. The following gradient was used: 0.0–2.0 min 100% B, 2.0–8.0 min gradual decrease to 50% B, 8.0–10.0 min 50% B, and at 10.0 min switch to 100% B and re-equilibration until 15 min. The injection volume was 5.0 μL, and the injector needle was washed for 5 s before each injection with acetonitrile/methanol/water 1:1:1 (v/v/v). Small molecules’ targeted data evaluation was carried out with the open-source software Skyline 20.1.0.76 [22].

GC–MS/MS measurement was performed to determine other intracellular metabolites that were not covered by HILIC-HRMS, adapting the procedure used by Mairinger et al. and Si-Hung et al. [23, 24]. In short, just in time online two-step derivatization was performed automatically using a Gerstel MPS2 dual-rail sample preparation robot (Gerstel GmbH, Muehlheim, Germany). Dried sample aliquots and standard mixtures containing equal amounts of the internal standard were mounted to the sample preparation robot and reconstituted using ethoxyamine hydrochloride in water-free pyridine. Samples and standards were incubated at 40 °C for 90 min for ethoximation, followed by silylation with N-methyl-N-(trimethylsilyl)trifluoroacetamide with 1% trimethylchlorosilane for 50 min. The derivatized samples and standards were kept at 4 °C for 5 min before automatic injection into the GC–MS/MS system. An Agilent Technologies 7010B GC–MS/MS Triple Quadrupole system (Waldbronn, Germany) equipped with electron ionization (EI) source and a deactivated nonpolar guard column (3 m × 0.25 mm I.D., Phenomenex) connected to a nonpolar Optima 1 MS Accent analytical column (60 m × 0.25 mm i.d., 0.25 μm film thickness, 100% dimethylpolysiloxane stationary phase) from Macherey–Nagel, Germany, was used. Helium was used as carrier gas at a constant flow rate of 1.3 mL min^−1^ and injection of 1.0-μL aliquots of sample solution was performed applying programmable temperature vaporization (PTV) (70 °C for 0.1 min; 12 °C min^−1^ to 260 °C, 1 min hold; 12 °C min^−1^ to 300 °C, 5 min hold). The following GC temperature gradient with a total cycle time of 33.2 min was used: 70 °C for 1 min, then gradual increase at 15 °C min^−1^ to 190 °C, at 5 °C min^−1^ to 225 °C, at 3 °C min^−1^ to 255 °C, and finally at 25 °C min^−1^ to 310 °C for 5 min. Information regarding all precursor and product ions along with collision energies used is provided in the supplementary material (Table S1). Data acquisition and evaluation were carried out with MassHunter Acquisition B.07.05.2479, MassHunter Quantitative QQQ B.10.00 (Agilent Technologies, CA, USA).

### Characterization and evaluation of reference material

In order to evaluate the in-house yeast-based reference material, various parameters were investigated in terms of homogeneity, stability, biological reproducibility, and interlaboratory comparison. Metabolomic assessment of six sample vials from the same fermentation batch was conducted via HILIC-HRMS and GC–MS/MS in order to evaluate homogeneity. In addition, one sample was injected three and four times to evaluate the technical repeatability of HILIC-HRMS and GC–MS/MS measurements, respectively. Dried aliquot extract stability at − 80 °C was assessed by subsequently thawing and measuring one sample vial from the same batch, thus spanning a period of 6 months. Three different fermentation batches were measured to figure out batch-to-batch biological reproducibility. Finally, an inter-method interlaboratory comparison was performed for selected metabolites based on samples from the same batch, with each laboratory using a different analytical method (i.e., one used HILIC-HRMS and the other GC–MS/MS).

## Results and discussion

### Characteristics of reference material

The candidate reference material was produced in independent and controlled fed-batch fermentations. Reproducible biomass growth rates were key for the reproducible in vivo synthesis of standards [25]. Next to the standardized fed-batch fermentation, standardized preparations in terms of rapid sampling, quenching, extraction, aliquoting of homogenous extracts, and evaporation of the extract were the basis for the preparation of the candidate reference material. The implemented procedures relied on previously established protocols [18]. In brief, cellular metabolism was quenched using cold methanol and cells were extracted using boiling ethanol followed by evaporation of the ethanolic extract.

**Fig. 1:**
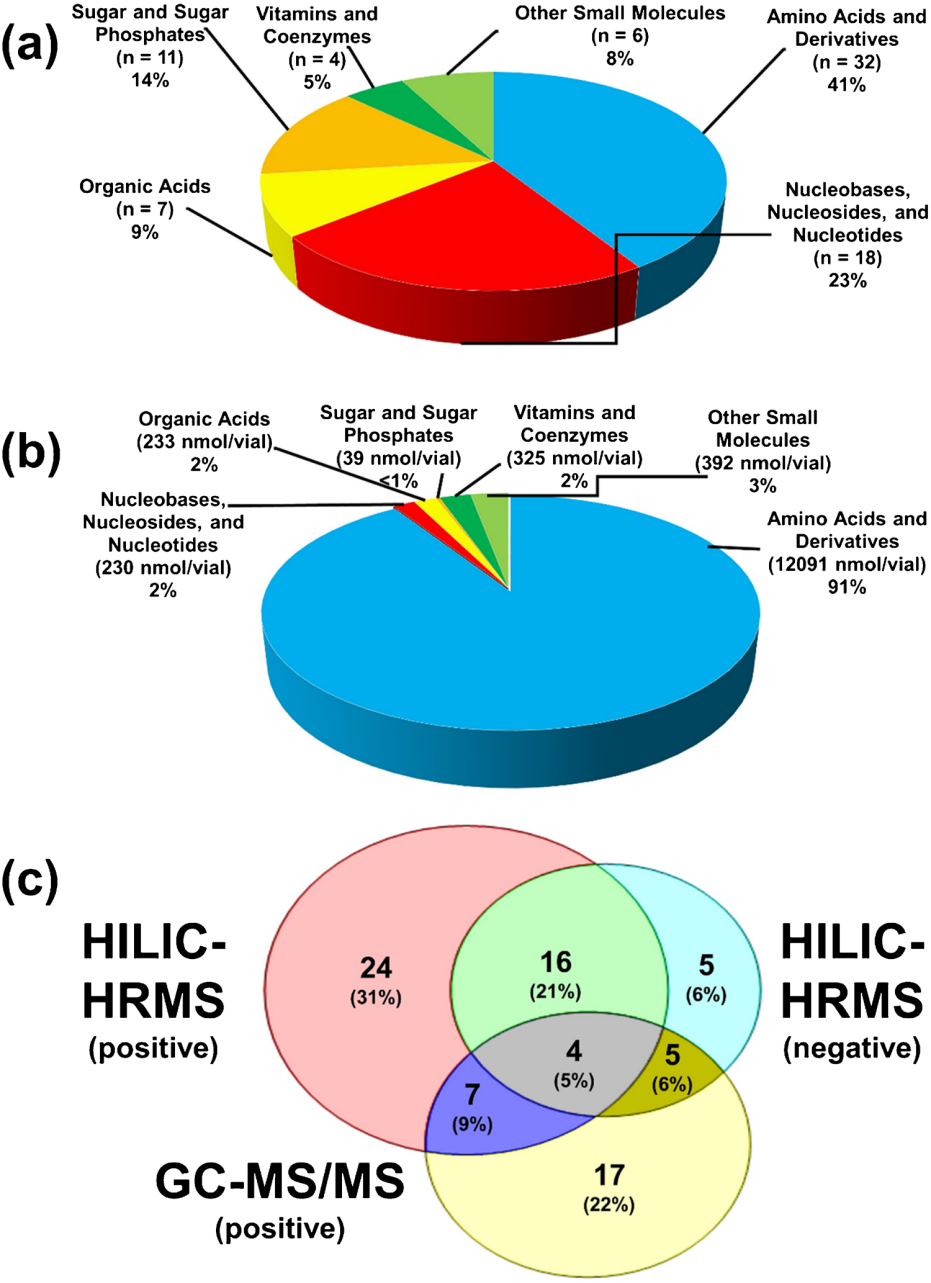
Overview and characteristics of in-house yeast-based reference material. **a** Distribution of analyzed metabolites based on the number of compounds detected from different metabolite classes, **b** distribution of the total concentration values (nmol vial^−1^) among metabolite classes, and **c** number of compounds detected depending on the analytical measurement platform. A total of 78 metabolites were determined using HILIC-HRMS and GC–MS/MS under positive and negative mode conditions

The dried aliquoted reference material extracts were reconstituted and determined by applying two complementary techniques, namely HILIC-HRMS and GC–MS/MS. As described elsewhere, a biotechnologically generated fully ^13^C-labeled *P. pastoris* cell extract was added to all samples and standards for internal standardization. Being derived from the same microorganism and produced by an equivalent fermentation using ^13^C-glucose as carbon source, the material offered the ideal match regarding both the composition and concentration ranges. The applied isotope dilution strategy (external calibration with ^13^C internal standards) enabled accurate quantification despite multiple sample preparation steps, as shown in different targeted metabolomics studies [23, 24, 26]. Volume losses during sample preparation or instrumental drifts could be compensated [18]. In this study, a portfolio of nearly 80 metabolites was absolutely quantified dominated by amino acids and derivatives followed by nucleobases, nucleosides, nucleotides, and other metabolites (Fig. 1). Sugars, sugar phosphates, vitamins, coenzymes, and other small molecules were included as well, demonstrating the material’s potential for comprehensive metabolomics studies. For biomarker discovery, biochemical pathway inspection, energy metabolism investigation, and other biological exploration in cellular samples, having access to absolute quantitative levels of a wide panel of metabolites, can be of decisive value [27]. Amino acids and their derivatives play an important role in the regulation of major metabolic pathways involved in protein synthesis. E.g., alanine (Ala), tryptophan (Trp), and aspartic acid (Asp) are conclusive monitoring parameters for the production of antibody fragments in *P. pastoris* [28]. Nucleobases, nucleosides, and nucleotides are next to constituting nucleic acids, involved in lipid and sugar metabolism, polyamine biosynthesis, purine, and pyrimidine biosynthesis, and serve as carriers for energy [29]. Organic acids, sugars, and sugar phosphates are significant compounds in the tricarboxylic acid (TCA) cycle, a central pathway in central carbon metabolism and energy generation [30]. The latter two are also building blocks for the backbone of DNA and RNA and substrates for the synthesis of polysaccharides and glycosides as well as in the interconversion of sugars [31]. Moreover, vitamins, coenzymes, and other small molecules participate in numerous metabolic and biochemical reactions [32]. More than 30 amino acids and their derivates and nearly 20 nucleobases, nucleosides, and nucleotides were determined via HILIC-HRMS and more than 10 sugars and sugar phosphates were analyzed via GC–MS/MS.

As can be observed in Fig. 1, the two instrumental platforms were complementary with regard to the target metabolites. GC–MS/MS followed by just in time two-step derivatization was ideally suited to absolutely quantify intermediates of the glycolysis and pentose phosphate pathway due to the unrivalled selectivity of the chromatographic separation allowing to separate sugar phosphate isomers. Supplementary Table S1–S2 detail the metabolite targets investigated by HILIC-HRMS and GC–MS/MS, respectively [24, 33, 34]. The technical uncertainty of the methods was on average < 10% (as exemplified with the uncertainty calculation for the amino acid threonine in the supplement); thus, the methods were fit for purpose.

**Fig. 2:**
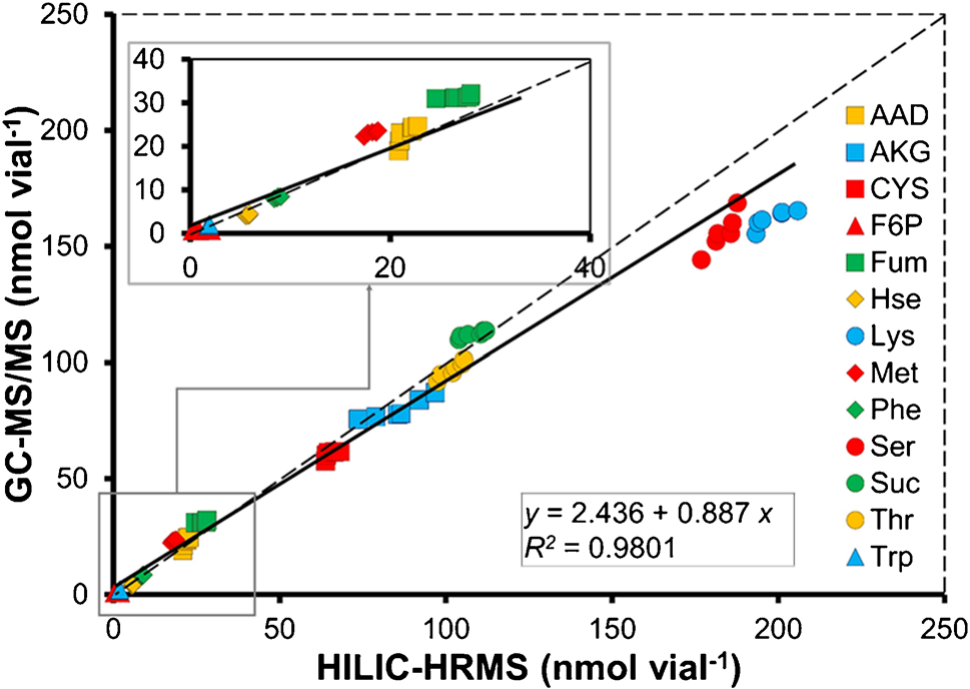
Weighted regression for intercomparison assessment of 13 selected metabolites from in-house yeast-based reference material measured with both HILIC-HRMS and GC–MS/MS methods in positive ionization mode. External calibration with internal standardization (fully ^13^C-labeled internal standard) was performed for each concentration (nmol vial^−1^). Reciprocal of the squared predicted values for standard deviation was used for weighting factors. The inset images showed the zoom-in regions of interest for clarity

A subset of metabolites was considered for the cross-validation of the two complementary methods and the calibrations established in the independent laboratories. Thirteen metabolites were amenable to both HILIC-HRMS and GC–MS/MS and were > LOQ in the yeast-derived material. Analysis was performed in parallel following one fermentation. For the majority of investigated compounds, the obtained concentration values were found to be in agreement (Fig. 2) proofing the methods fit for purpose and the overall validity of the study.

### Evaluation of reference material

**Fig. 3:**
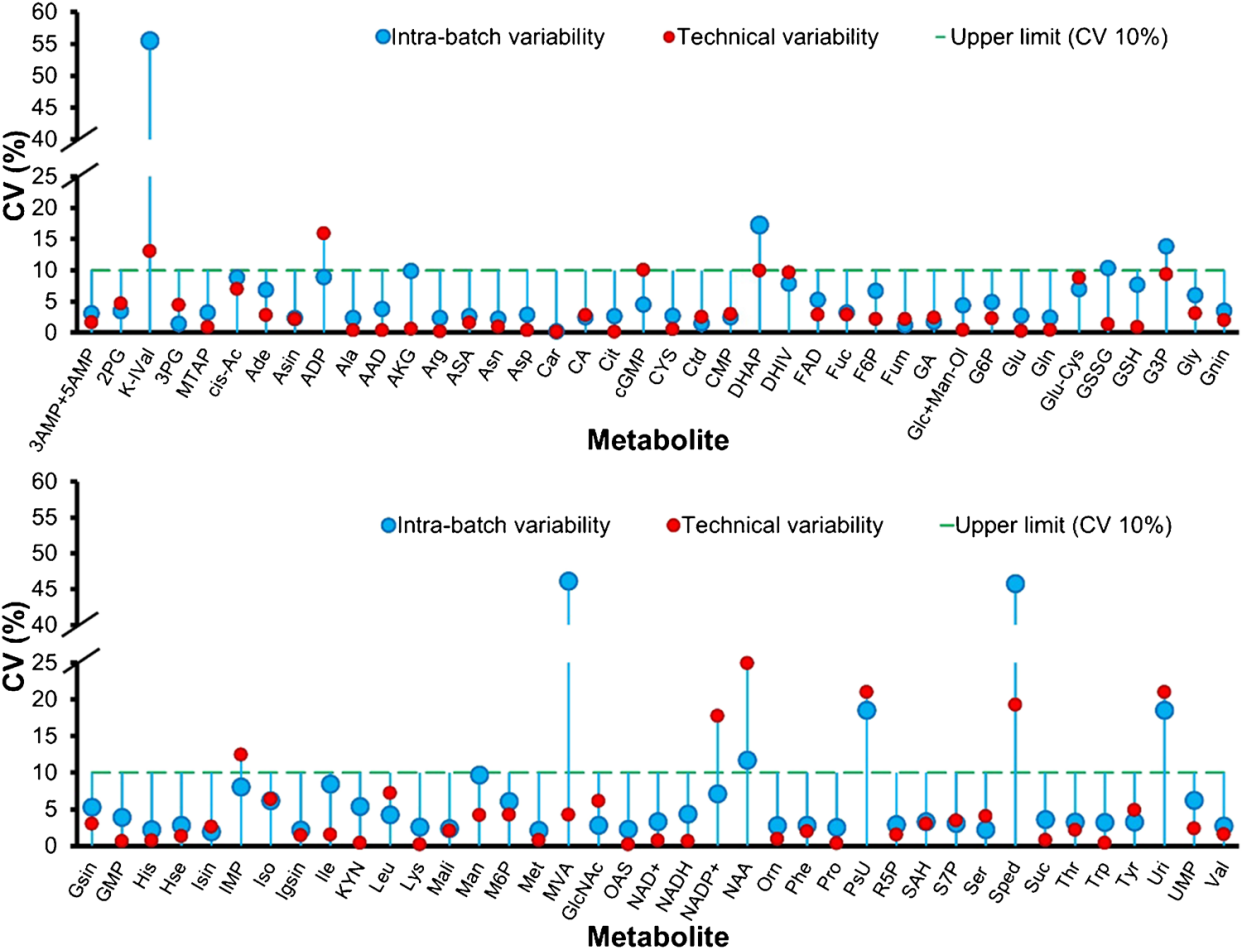
Homogeneity assessment of in-house yeast-based reference material for 78 investigated metabolites. Metabolites were measured using HILIC-HRMS and GC–MS/MS under positive and negative mode conditions. Quantification was based on external calibration with the addition of a fully ^13^C-labeled internal standard. For the individual metabolite, the black-filled circle represents the variability (% CV) of concentration values among different vials from the same batch (*n* = 6), and the unfilled circle represents technical variability of the measurement based on three consecutive injections from the same vial (*n* = 3) for LC–MS and four injections for GC–MS/MS (*n* = 4). The dashed line indicates the upper limit variability (CV 10%)

In order to evaluate within-batch homogeneity among the vials, a set of (*n* = 6) dried aliquots per batch were assessed by replicate measurement on the two platforms. Figure 3 plots the technical repeatability versus the within-batch repeatability for the targets. For the subset of metabolites which were amenable to HILIC-HRMS and GC–MS/MS, the quantitative value obtained by the method with the superior technical repeatability was plotted. The coefficient of variation (CV) for intra-batch variability was excellent, ranging below 10% for more than 80% of the observed metabolites, and was in the range of the technical variability (Fig. 3, Supplementary Table S3). Amino acids and their derivatives showed even < 3% CV on average, reflecting excellent homogeneity. Only a minor fraction of metabolites revealed CVs above 45%, such as spermidine (Sped), mevalonic acid (MVA), and 3-methyl-2-oxovaleric acid (K-IVal). While amino acids and derivates were in the range of 0.5 to 3000 nmol vial^−1^, metabolites showing such a high CV were dominated by low intracellular abundance resulting in poor technical repeatability and thus apparent poor homogeneity [35].

**Fig. 4:**
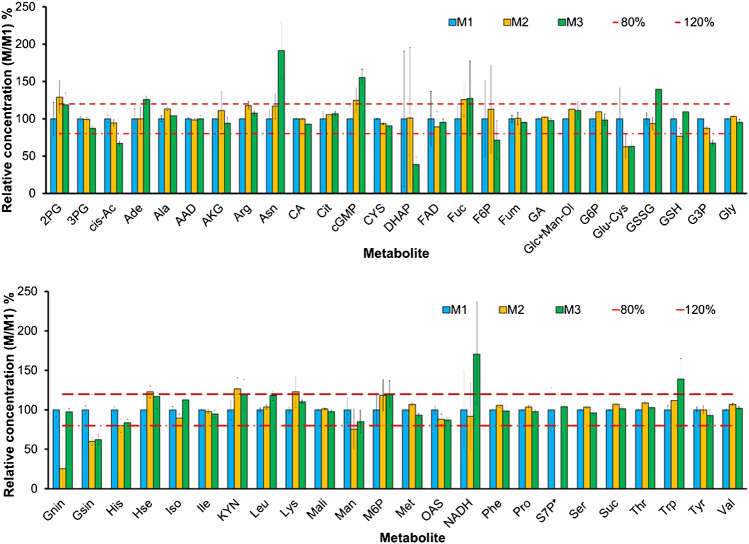
Stability assay for 50 selected metabolites from in-house yeast-based reference material within 6 months of time. Investigated metabolites were measured by HILIC UPLC-Orbitrap MS and GC–MS/MS under positive mode conditions. Yeast-based reference material was stored at − 80 ℃ before measurement. A fully ^13^C-labeled internal standard was added to the samples and external calibrants for metabolite quantitation. Relative concentration was calculated as the concentration value obtained in each measurement M1 (0 months), M2 (3 months), and M3 (6 months), in nmol vial^−1^ divided by the concentration value obtained from the first measurement (M1) and given in percent. (*) Data not included due to outlier data sedoheptulose-7-phosphate (S7P) for measurement M2. Error bars describe the standard deviation of six replicate measurements (*n* = 6) from the same batch. Dotted and dashed lines represent 80% and 120% relative concentration, respectively

Next, storage of dried yeast extracts at − 80 ℃ was investigated for 3 different time points over a period of 6 months. Figure 4 and Supplementary Table S4 give the obtained data. As can be readily observed, the stability was suited for a reference material, as the major fraction of metabolite showed only minor concentration (< 20%) differences upon storage. A slightly higher concentration decrease was observed for a small number of sugars and sugar phosphates such as glycerol-3-phosphosphate (G3P) and mannose (Man).

**Fig. 5:**
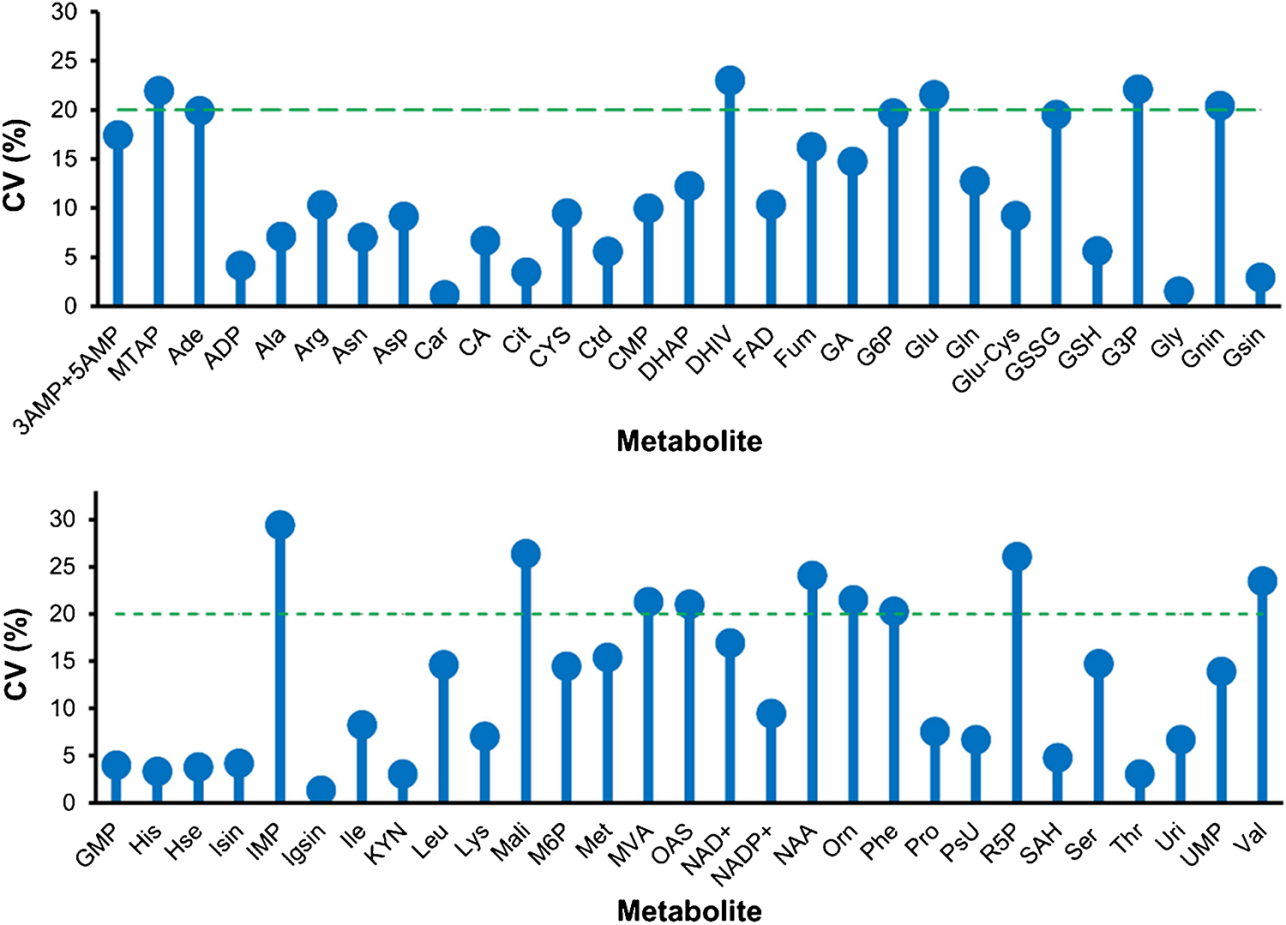
Typical inter-batch biological reproducibility of selected metabolites from in-house yeast-based reference material. Selected metabolites were quantitatively analyzed by HILIC-HRMS and GC–MS/MS under positive mode conditions. External calibration with the addition of a fully ^13^C-labeled internal standard was performed for metabolite measurement. The graph shows relative standard deviations (% CV) of metabolites between the different batches (*n* = 3). The CV calculation is based on the mean values obtained from six vials from each batch. The dashed line indicates a CV of 20%

Finally, in order to proof the concept of producing reference material on demand, the assessment of inter-batch biological reproducibility was essential. Based on homogeneity and stability data, a panel of 50 metabolites were selected and investigated in 3 independent fermentations (6 six replicate samples of each batch). The assessed analytical figures of merit were excellent. Inter-batch variation (CV) was < 20% for approximately 50% of the targets (Fig. 5). Exceptional biological reproducibility was achieved for carnitine (Car), isoguanosine (Igsin), guanosine (Gsin), and glycine (Gly) with variations (CV) < 3%. The amino acid concentrations obtained upon replicative fermentation were associated with % CV which were comparable or even lower to the % CV of the certified amino acid concentrations in SRM 1950 [36]. In contrast, a few compounds, such as mannose (Man), fructose (Fuc), and succinic acid (Suc), showed high variations > 60% (Supplementary Fig. S1). Reference samples across multiple batches in terms of quality control samples for clinical non-targeted metabolomics ideally provide control ranges of 30% [37]. However, certified and indicative values in state-of-the-art metabolomics biological reference material only partly fulfill this quality criterion [10, 17, 38]. The internationally accepted consensus values for lipid species obtained for SRM1950 are often associated with significantly higher variations [10, 12, 16, 38]. Thus, the proposed strategy could overcome the problem of providing large batches of biological matrix from the start phase of material production delivering quantitative values on demand with control ranges comparable to the state of the art.

## Conclusion

The results of the current study indicate that both reproducibility and stability were fit for purpose for a major part of yeast extract metabolites investigated, supporting its potential as a reference material for quality control in metabolomics studies. Regarding the proposed strategy of in vivo synthesis, the study was promising, showing the potential of biological reference materials’ production on demand. The yeast material has already been proposed as benchmarking library and thus quality control for non-targeted metabolomics and lipidomics. In this work, this concept was expanded towards absolute quantification. We showed that it is possible to produce biomass controlling the metabolome pool sizes. The proposed reference material is comprable to the state-of-the-art materials but offers higher throughput and cost-effectiveness in the production. Further assessment with orthogonal methods and collaborative studies will be required to increase metabolite coverage and continue the certification campaign in the future.

## Supplementary Information

Below is the link to the electronic supplementary material.

Supplementary file1 (PDF 313 KB)
