# Supplementary material for: Yeast-based reference materials for quantitative metabolomics
Source: Anal Bioanal Chem. 2021 Oct 13;414(15):4359–68. doi: 10.1007/s00216-021-03694-w (PMC9142427; doi:10.1007/s00216-021-03694-w)
Supplement: Supplementary file 1 — Supplementary file1 (PDF 313 KB) [file 216_2021_3694_MOESM1_ESM.pdf]

Supporting Information for

Yeast based reference materials for quantitative metabolomics

Hendri Wasito[a,b], Gerrit Hermann[c,d], Veronika Fitz[e], Christina Troyer[a], Stephan Hann[a], Gunda Koellensperger[e,f *]

[a] Institute of Analytical Chemistry, Department of Chemistry, University of Natural Resources and Life Sciences (BOKU) Vienna, Muthgasse 18, 1190 Vienna, Austria.
[b] Department of Pharmacy, Faculty of Health Sciences, Jenderal Soedirman University, Dr. Soeparno Street, 53122 Purwokerto, Indonesia.
[c]Core Facility Mass Spectrometry, University of Natural Resources and Life Sciences (BOKU) Vienna, Muthgasse 11, 1190 Vienna, Austria.
[d] ISOtopic solutions, Waehringer Str. 38, 1090 Vienna, Austria
[e] Department of Analytical Chemistry, Faculty of Chemistry, University of Vienna, Waehringer Str. 38, 1090 Vienna, Austria
[f] Vienna Metabolomics Center (VIME), University of Vienna, Althanstraße 14, 1090 Vienna, Austria

Correspondence to be addressed to:
Gunda Koellensperger
Department of Analytical Chemistry, Faculty of Chemistry, University of Vienna, Waehringer Str. 38, 1090 Vienna, Austria.
E-mail: gunda.koellensperger@univie.ac.at

Table of contents:

Table S1. Summary of monitored GC-MS/MS transition ion parameters in positive mode for investigated metabolites.

Table S2. Summary of monitored HILIC-HRMS accurate masses for the investigated metabolites.

Table S3. Multicomponent homogeneity of in-house yeast-based reference materials.

Table S4. Stability data from some selected metabolites of in-house yeast-based reference materials.

Table S5. Inter-batch biological reproducibility from some selected metabolites of in-house yeast-based reference materials.

Table S6. In-house yeast-based standard reference materials interlaboratory comparison from some selected metabolites.

Table S7. Characterization and quantification of uncertainty source for the preparation of standard calibration for threonine quantification in reference material

Figure of contents:

Fig. S1. Inter-batch biological reproducibility for in-house yeast-based standard reference materials.

Fig. S2. Variability comparison between biological reproducibility and homogeneity for in-house yeast-based standard reference materials.

Table S1. Summary of monitored GC-MS/MS transition ion parameters in positive mode for investigated metabolites. Fully ^{13}C-labeled yeast extract was used as an internal standard.

Abbr.	Compound	Segment	Precursor ion		Product ion		RT (min)	Coll. E. (eV)
			Target	ISTD	Target	ISTD		
2PG	2-Phosphoglyceric acid	10	459.1	462.1	299.1	299.1	16.81	10
3PG	3-Phosphoglyceric acid	10	459.1	462.1	299.1	299.1	17.21	10
cis-Ac	Aconitic acid	9	375.1	381.1	285.1	291.1	16.16	5
AAD	alpha-Aminoadipic acid	8	362.2	368.2	244.1	249.1	15.87	10
AKG	alpha-Ketoglutaric acid	7	288.1	293.1	154	158	14.37	15
Asp	Aspartic acid	6	232.1	338.1	188.1	147.1	13.44	10
CA	Citric acid	11	273.1	278.1	229.1	233.1	17.55	10
CYS	Cystathionine	16	278.2	282.2	160.2	163.2	24.67	10
DHAP	Dihydroxyacetone phosphate	10	414.1	417.2	299.1	372.1	16.88	10
Fuc	Fructose	12	217.1	220.1	101	103	19.56	15
F6P	Fructose-6-phosphate	17	459.2	462.2	315.1	299.1	27.66	15
Fum	Fumaric acid	3	245.1	249.1	73.1	73.1	11.45	20
Gal	Glactose	13	319.2	323.2	229.1	233.1	19.99	10
GA	Gluconic acid	14	333.1	337.1	143	146	21.35	15
Glc+Man-Ol	Glucose+Mannitol	13	319.2	323.2	229.1	233.1	20.14	10
G6P	Glucose-6-phosphate	17	471.2	475.2	387.1	387.1	27.92	15
Glu	Glutamic acid	7	246.1	250.1	156.1	160.1	14.63	10
G3P	Glycerol-3-phosphosphate	10	445.2	448.2	299.1	299.1	16.62	15
Gly	Glycine	3	276.1	278.1	248.1	249.1	11.28	10
Hse	Homoserine	5	320.2	324.2	230.1	147.1	12.67	20
Iso	Isocitric acid	11	273.1	278.1	83	87	17.44	10
Lys	Lysine	12	434.2	322.2	156.1	161.1	19.39	5
Mali	Malic acid	5	335.1	339.1	147.1	147.1	13.01	10
Man	Mannose	13	319.2	323.2	229.1	233.1	19.9	10
M6P	Mannose-6-phosphate	17	471.2	475.2	387.1	387.1	28.21	15
Met	Methionine	6	176.1	180.1	128.1	131.1	13.45	10
GlcNAc	Phenylalanine	7	294.1	303.1	147.1	147.1	14.73	5
R5P	Ribose-5-phosphate	15	459.2	462.2	315.1	315.1	23.85	10
S7P	Sedoheptulose-7-phosphate	19	471.2	475.2	387.1	387.1	30.88	10
Ser	Serine	4	306.1	309.2	278.1	280.2	11.71	5
Suc	Succinic acid	3	247.1	251.1	147.1	73.1	11.07	30
Thr	Threonine	4	320.2	324.2	147.1	147.1	12.04	20
Trp	Tryptophan	16	202.1	211.1	73.1	73.1	24.68	15
Val	Valine	3	218.1	220.1	100.1	101.1	10.3	5

Table S2. Summary of monitored HILIC-HRMS accurate masses for investigated metabolites. $[M+H]^+$ and $[M-H]^-$ were monitored in positive and negative polarities, respectively. Fully ^{13}C-labeled yeast extract was used as an internal standard.

Abbr.	Compound	Positive			Negative		
		Target	ISTD	RT (min)	Target	ISTD	RT (min)
3AMP+ 5AMP	3'-Adenosine monophosphate+ 5'-Adenosine monophosphate	348.0704	358.1039	6.37	346.0558	356.0894	6.64
K-IVal	3-Methyl-2-oxovaleric acid	-	-	-	129.0557	135.0758	1.89
MTAP	5'-Deoxy-5'-Methylthioadenosine	298.0968	309.1337	1.71	-	-	-
cis-Ac	Aconitic acid	-	-	-	173.0092	179.0293	2.66
Ade	Adenine	136.0618	141.0785	2.19	134.0472	139.064	2.26
Asin	Adenosine	268.104	278.1376	2.30	-	-	-
ADP	Adenosine diphosphate	428.0367	438.0702	7.89	-	-	-
Ala	Alanine	90.055	93.065	5.11	-	-	-
AAD	alpha-Aminoadipic acid	162.0761	168.0962	5.72	160.0615	166.0817	6.02
AKG	alpha-Ketoglutaric acid	-	-	-	145.0142	150.031	4.20
Arg	Arginine	175.119	181.1391	6.46	173.1044	179.1245	6.84
ASA	Argininosuccinic acid	291.1299	301.1635	7.43	289.1154	299.1489	7.68
Asn	Asparagine	133.0608	137.0742	5.87	131.0462	135.0596	6.23
Asp	Aspartic acid	134.0448	138.0582	6.52	132.0302	136.0437	6.78
Car	Carnitine	162.1125	169.136	3.81	-	-	-
Cit	Citrulline	176.103	182.1231	5.94	174.0884	180.1085	6.30
cGMP	Cyclic guanosine monophosphate	346.0547	356.0883	5.40	-	-	-
CYS	Cystathionine	223.0747	230.0982	7.30	221.0602	228.0836	7.53
Ctd	Cytidine	244.0928	253.123	3.20	-	-	-
CMP	Cytidine monophosphate	324.0591	333.0893	6.96	322.0446	331.0748	7.27
DHIV	Dihydroxyisovaleric acid	-	-	-	133.0506	138.0674	3.90
FAD	Flavin adenin dinucleotide	786.1644	813.255	6.73	-	-	-
F6P	Fructose-6-phosphate	-	-	-	259.0224	265.0426	7.39
Fum	Fumaric acid	-	-	-	115.0037	119.0171	4.09
Glu	Glutamic acid	148.0604	153.0772	6.07	146.0619	151.0627	6.35
Gln	Glutamine	147.0764	152.0932	5.73	145.0619	150.0786	6.10
Glu-Cys	Glutamyl-cysteine	251.0696	259.0965	6.35	-	-	-
GSSG	Glutathione, oxidized	613.1592	637.2263	8.07	611.1447	631.2118	8.26
GSH	Glutathione, reduced	308.0911	318.1246	6.37	306.0765	316.1101	6.65
Gnin	Guanine	152.0567	157.0735	2.79	-	-	-
Gsin	Guanosine	284.0989	294.1325	3.34	-	-	-
GMP	Guanosine monophosphate	364.0653	374.0988	6.97	362.0507	372.0843	7.26
His	Histidine	156.6768	162.0969	6.47	154.0622	160.0823	6.84
Hse	Homoserine	120.0655	124.0789	5.42	-	-	-
Isin	Inosine	269.088	279.1216	2.80	-	-	-
IMP	Inosine monophosphate	349.0544	359.0879	6.57	-	-	-
Igsin	Isoguanosine	284.0989	294.1325	3.34	-	-	-
Ile	Isoleucine	132.1019	138.122	3.12	-	-	-

KYN	Kynurenine	209.0921	219.1256	2.84	-	-	-
Leu	Leucine	132.1019	138.122	2.93	-	-	-
Lys	Lysine	147.1128	153.1329	6.65	-	-	-
Met	Methionine	150.0583	155.0759	3.40	-	-	-
MVA	Mevalonic acid	-	-	-	147.0663	153.0864	2.08
OAS	N-Acetylserine	148.0604	153.0772	6.07	-	-	-
NAD+	NAD+	664.1164	685.1869	7.14	662.6018	683.1723	7.44
NADH	NADH	666.132	687.2025	6.86	664.1175	685.1879	7.16
NADP+	NADP+	744.0827	765.1532	7.96	-	-	-
NAA	Nicotinamide	123.0553	129.0754	1.78	-	-	-
Orn	Ornithine	133.0972	138.1139	6.69	-	-	-
Phe	Phenylalanine	166.0863	175.1164	2.82	-	-	-
Pro	Proline	116.0706	121.0874	4.01	-	-	-
PsU	Pseudouridine	-	-	-	243.0623	252.0925	3.63
R5P	Ribose-5-phosphate	-	-	-	229.0119	234.0287	6.70
SAH	S-Adenosyl-L-homocysteine	385.1289	399.1758	5.80	-	-	-
Ser	Serine	106.0499	109.0599	5.80	-	-	-
Sped	Spermidine	146.1652	153.1887	7.05	-	-	-
Suc	Succinic acid	-	-	-	117.0193	121.0328	2.00
Thr	Threonine	120.1655	124.0789	5.33	-	-	-
Trp	Tryptophan	205.0972	216.1341	2.72	-	-	-
Tyr	Tyrosine	182.0812	191.1114	3.78	-	-	-
Uri	Uridine	-	-	-	243.0623	256.0925	3.63
UMP	Uridine monophosphate	325.0431	334.0733	6.42	323.0286	332.0588	6.72

Table S3. Multicomponent homogeneity of in-house yeast-based reference materials. (n = 6 for intra-batch measurement; n = 3 and n = 4 for HILIC-HRMS and GC-MS/MS technical replicate measurement, respectively).

Abbr.	Compound	Polarity	Method	Concentration Average (nmol vial^{-1})	Concentration CV (%)	Technical Replicate CV (%)
3AMP+ 5AMP	3'-Adenosine monophosphate +5'-Adenosine monophosphate	Negative	HILIC-HRMS	80.99	3.09	1.66
2PG	2-Phosphoglyceric acid	Positive	GC-MS/MS	0.17	3.45	4.63
K-IVal	3-Methyl-2-oxovaleric acid	Negative	HILIC-HRMS	4.41	55.48	13.07
3PG	3-Phosphoglyceric acid	Positive	GC-MS/MS	1.01	1.41	4.44
MTAP	5'-Deoxy-5'-Methylthioadenosine	Positive	HILIC-HRMS	4.00	3.23	0.87
cis-Ac	Aconitic acid	Positive	GC-MS/MS	0.45	8.84	6.97
Ade	Adenine	Positive	HILIC-HRMS	0.42	6.86	2.78
Asin	Adenosine	Positive	HILIC-HRMS	1.36	2.30	2.15
ADP	Adenosine diphosphate	Positive	HILIC-HRMS	87.19	8.88	15.88
Ala	Alanine	Positive	HILIC-HRMS	1051.65	2.31	0.34
AAD	alpha-Aminoadipic acid	Negative	HILIC-HRMS	21.64	3.78	0.37
AKG	alpha-Ketoglutaric acid	Negative	HILIC-HRMS	85.51	9.87	0.60
Arg	Arginine	Positive	HILIC-HRMS	1161.73	2.30	0.16
ASA	Argininosuccinic acid	Positive	HILIC-HRMS	23.16	2.60	1.58
Asn	Asparagine	Positive	HILIC-HRMS	102.72	2.22	0.93
Asp	Aspartic acid	Positive	HILIC-HRMS	1627.90	2.83	0.37
Car	Carnitine	Positive	HILIC-HRMS	0.90	0.16	0.11
CA	Citric acid	Positive	GC-MS/MS	22.57	2.47	2.77
Cit	Citrulline	Positive	HILIC-HRMS	93.96	2.64	0.15
cGMP	Cyclic guanosine monophosphate	Positive	HILIC-HRMS	0.44	4.50	10.06
CYS	Cystathionine	Positive	HILIC-HRMS	65.65	2.70	0.56
Ctd	Cytidine	Positive	HILIC-HRMS	0.14	1.44	2.53
CMP	Cytidine monophosphate	Positive	HILIC-HRMS	8.96	2.42	2.95
DHAP	Dihydroxyacetone phosphate	Positive	GC-MS/MS	0.04	17.20	9.90
DHIV	Dihydroxyisovaleric acid	Negative	HILIC-HRMS	9.51	7.84	9.64
FAD	Flavin adenin dinucleotide	Positive	HILIC-HRMS	1.54	5.20	2.85
Fuc	Fructose	Positive	GC-MS/MS	4.58	3.21	2.87
F6P	Fructose-6-phosphate	Positive	GC-MS/MS	0.57	6.66	2.14
Fum	Fumaric acid	Positive	GC-MS/MS	31.38	1.19	2.17
GA	Gluconic acid	Positive	GC-MS/MS	3.26	1.69	2.40
Glc+Man -Ol	Glucose+Mannitol	Positive	GC-MS/MS	11.50	4.33	0.40
G6P	Glucose-6-phosphate	Positive	GC-MS/MS	1.54	4.90	2.28
Glu	Glutamic acid	Negative	HILIC-HRMS	2889.74	2.69	0.22
Gln	Glutamine	Positive	HILIC-HRMS	748.68	2.41	0.43
Glu-Cys	Glutamyl-cysteine	Positive	HILIC-HRMS	4.88	6.94	8.76
GSSG	Glutathione, oxidized	Positive	HILIC-HRMS	106.02	10.33	1.34
GSH	Glutathione, reduced	Positive	HILIC-HRMS	257.31	7.65	0.86
G3P	Glycerol-3-phosphosphate	Positive	GC-MS/MS	12.70	13.78	9.34
Gly	Glycine	Positive	GC-MS/MS	63.66	5.98	3.10

Gnin	Guanine	Positive	HILIC-HRMS	0.15	3.50	1.97
Gsin	Guanosine	Positive	HILIC-HRMS	0.19	5.27	3.01
GMP	Guanosine monophosphate	Positive	HILIC-HRMS	13.18	3.88	0.62
His	Histidine	Positive	HILIC-HRMS	87.77	2.23	0.73
Hse	Homoserine	Positive	HILIC-HRMS	5.73	2.81	1.33
Isin	Inosine	Positive	HILIC-HRMS	0.23	1.92	2.61
IMP	Inosine monophosphate	Positive	HILIC-HRMS	0.82	8.03	12.44
Iso	Isocitric acid	Positive	GC-MS/MS	0.72	6.15	6.34
Igsin	Isoguanosine	Positive	HILIC-HRMS	0.34	2.18	1.43
Ile	Isoleucine	Positive	HILIC-HRMS	15.96	8.46	1.52
KYN	Kynurenine	Positive	HILIC-HRMS	0.84	5.36	0.36
Leu	Leucine	Positive	HILIC-HRMS	12.94	4.21	7.22
Lys	Lysine	Positive	HILIC-HRMS	198.33	2.55	0.17
Mali	Malic acid	Positive	GC-MS/MS	120.25	2.35	2.06
Man	Mannose	Positive	GC-MS/MS	0.90	9.65	4.21
M6P	Mannose-6-phosphate	Positive	GC-MS/MS	0.42	6.05	4.24
Met	Methionine	Positive	GC-MS/MS	23.13	2.10	0.79
MVA	Mevalonic acid	Negative	HILIC-HRMS	6.84	46.13	4.25
OAS	N-Acetylserine	Positive	HILIC-HRMS	2669.10	2.26	0.18
NAD+	NAD+	Negative	HILIC-HRMS	36.58	3.31	0.74
NADH	NADH	Positive	HILIC-HRMS	278.00	4.33	0.63
NADP+	NADP+	Positive	HILIC-HRMS	3.37	7.10	17.71
NAA	Nicotinamide	Positive	HILIC-HRMS	6.77	11.68	24.92
Orn	Ornithine	Positive	HILIC-HRMS	487.19	2.75	0.92
Phe	Phenylalanine	Positive	HILIC-HRMS	8.78	2.78	1.98
Pro	Proline	Positive	HILIC-HRMS	239.93	2.49	0.31
PsU	Pseudouridine	Negative	HILIC-HRMS	0.87	18.51	20.97
R5P	Ribose-5-phosphate	Positive	GC-MS/MS	16.40	2.91	1.54
SAH	S-Adenosyl-L-homocysteine	Positive	HILIC-HRMS	2.29	3.29	3.00
S7P	Sedoheptulose-7-phosphate	Positive	GC-MS/MS	1.68	3.03	3.43
Ser	Serine	Positive	HILIC-HRMS	183.18	2.20	4.02
Sped	Spermidine	Positive	HILIC-HRMS	3.77	45.74	19.27
Suc	Succinic acid	Negative	HILIC-HRMS	108.64	3.00	0.80
Thr	Threonine	Positive	HILIC-HRMS	101.79	3.24	2.17
Trp	Tryptophan	Positive	HILIC-HRMS	1.82	3.22	0.40
Tyr	Tyrosine	Positive	HILIC-HRMS	6.47	3.24	4.86
Uri	Uridine	Negative	HILIC-HRMS	0.87	18.51	20.97
UMP	Uridine monophosphate	Negative	HILIC-HRMS	28.20	6.19	2.36
Val	Valine	Positive	GC-MS/MS	60.41	2.70	1.58

Table S4. Stability data from some selected metabolites of in-house yeast-based reference materials. Three different time measurements were carried out within 6 months, with six replicates quantification for each metabolite (n = 6).

Compound	Method	0 months		3 months		6 months	
		Average (nmol vial^{-1})	CV (%)	Average (nmol vial^{-1})	CV (%)	Average (nmol vial^{-1})	CV (%)
2PG	GC-MS	0.06	21.86	0.07	17.48	0.07	13.34
3PG	GC-MS/MS	0.55	2.14	0.55	2.32	0.48	2.79
cis-Ac	GC-MS/MS	1.61	4.78	1.52	5.28	1.08	4.64
Ade	HILIC-HRMS	0.46	13.07	0.46	15.28	0.58	3.79
Ala	HILIC-HRMS	902.32	3.83	1021.94	2.11	940.57	2.93
AAD	GC-MS/MS	16.82	1.42	16.60	1.39	16.81	4.81
AKG	GC-MS/MS	30.94	21.20	34.43	22.27	29.16	7.54
Arg	HILIC-HRMS	881.55	6.80	1037.72	4.90	949.28	3.35
Asn	HILIC-HRMS	115.94	12.81	135.70	14.42	222.17	19.65
CA	GC-MS/MS	27.17	1.06	27.07	1.33	25.21	1.34
Cit	HILIC-HRMS	86.13	10.55	90.86	13.09	92.05	3.33
cGMP	HILIC-HRMS	0.27	17.07	0.33	13.87	0.41	7.17
CYS	GC-MS/MS	66.38	0.96	62.02	1.41	60.22	1.84
DHAP	GC-MS/MS	0.10	90.77	0.10	92.72	0.04	26.92
FAD	HILIC-HRMS	1.57	36.84	1.41	22.48	1.50	4.70
Fuc	GC-MS/MS	0.83	19.61	1.05	18.14	1.06	39.32
F6P	GC-MS/MS	0.35	51.12	0.40	52.61	0.25	36.84
Fum	GC-MS/MS	27.95	4.88	28.11	8.22	26.63	7.16
GA	GC-MS/MS	2.66	2.72	2.72	0.78	2.60	2.11
Glc+Man-Ol	GC-MS/MS	5.90	7.54	6.67	8.89	6.57	11.18
G6P	GC-MS/MS	1.20	10.40	1.32	14.03	1.18	7.82
Glu-Cys	HILIC-HRMS	3.00	41.71	1.88	26.28	1.89	11.30
GSSG	HILIC-HRMS	58.79	9.08	55.21	9.27	81.96	8.43
GSH	HILIC-HRMS	172.75	15.98	132.66	13.74	189.33	6.62
G3P	GC-MS/MS	29.69	3.75	25.94	3.15	20.07	5.23
Gly	GC-MS/MS	64.91	0.47	67.12	0.72	61.96	3.91
Gnin	HILIC-HRMS	0.11	9.10	0.03	155.64	0.11	4.14
Gsin	HILIC-HRMS	0.28	5.15	0.17	8.72	0.18	10.84
His	HILIC-HRMS	99.00	3.55	79.07	10.89	82.91	5.31
Hse	GC-MS/MS	5.18	8.38	6.37	6.01	6.07	13.56
Iso	GC-MS/MS	0.91	4.33	0.82	5.85	1.03	4.00
Ile	GC-MS/MS	15.59	1.10	15.25	2.27	14.75	5.51
KYN	HILIC-HRMS	0.25	12.62	0.32	11.01	0.30	15.88
Leu	GC-MS/MS	8.11	3.10	8.42	3.02	9.59	5.48
Lys	HILIC-HRMS	122.79	3.92	151.08	16.39	135.31	2.01
Mali	GC-MS/MS	80.47	1.21	81.24	1.19	78.86	2.04
Man	GC-MS/MS	0.32	16.43	0.25	34.02	0.28	16.66
M6P	GC-MS/MS	0.28	19.82	0.33	16.94	0.34	14.41
Met	GC-MS/MS	15.75	1.86	16.85	2.77	14.69	2.44
OAS	HILIC-HRMS	2192.49	5.42	1930.60	7.55	1906.06	3.15
NADH	HILIC-HRMS	102.54	47.63	94.19	46.91	174.75	38.72
Phe	GC-MS/MS	6.48	1.76	6.83	2.76	6.39	8.28

Pro	GC-MS/MS	298.40	0.98	309.56	1.16	292.40	2.08
S7P*	GC-MS/MS	0.43	27.93	-	-	0.45	15.78
Ser	GC-MS/MS	152.53	1.14	157.95	1.25	146.67	3.03
Suc	GC-MS/MS	20.40	0.78	21.89	1.22	20.71	1.82
Thr	GC-MS/MS	101.18	1.03	109.98	1.22	104.15	1.59
Trp	HILIC-HRMS	0.93	4.97	1.05	9.15	1.30	18.64
Tyr	HILIC-HRMS	3.87	3.37	3.87	5.57	3.60	8.59
Val	GC-MS/MS	40.43	0.84	43.25	1.00	41.20	2.21

Note: *Data not included due to outlier data Sedoheptulose-7-phosphate (S7P) for measurement 2. All measurements were performed in positive mode.

Table S5. Inter-batch biological reproducibility from some selected metabolites of in-house yeast-based reference materials. Six replicate measurements were performed for evaluating three different inter-batch variabilities (n = 3).

Compound	Polarity	Method	Average Concentration (nmol vial^{-1})			Inter-batch CV (%)
			1st batch	2nd batch	3rd batch	
AMP+AMP	Negative	HILIC-HRMS	98.17	69.47	80.99	17.43
2PG	Positive	GC-MS/MS	0.07	0.15	0.17	41.88
K-IVal	Negative	HILIC-HRMS	4.38	1.55	4.41	47.66
3PG	Positive	GC-MS/MS	0.48	1.04	1.01	37.46
MTAP	Positive	HILIC-HRMS	2.68	4.08	4.00	21.95
cis-Ac	Negative	HILIC-HRMS	0.52	0.29	0.27	38.88
Ade	Positive	HILIC-HRMS	0.33	0.50	0.42	19.89
Asin	Positive	HILIC-HRMS	2.40	1.43	1.36	33.48
ADP	Positive	HILIC-HRMS	80.32	84.71	87.19	4.14
Ala	Positive	HILIC-HRMS	922.80	1039.59	1051.65	7.08
AAD	Positive	GC-MS/MS	16.81	34.27	22.67	36.15
AKG	Positive	GC-MS/MS	29.16	34.57	79.91	58.21
Arg	Positive	HILIC-HRMS	1003.58	957.12	1161.73	10.31
ASA	Positive	HILIC-HRMS	12.04	18.15	23.16	31.32
Asn	Positive	HILIC-HRMS	112.16	97.79	102.72	7.00
Asp	Positive	HILIC-HRMS	1932.76	1695.58	1627.90	9.14
Car	Positive	HILIC-HRMS	0.91	0.89	0.90	1.13
CA	Positive	GC-MS/MS	25.21	22.44	22.57	6.68
Cit	Positive	HILIC-HRMS	87.69	90.83	93.96	3.45
cGMP	Positive	HILIC-HRMS	0.26	0.15	0.44	51.33
CYS	Positive	HILIC-HRMS	65.21	55.27	65.65	9.46
Ctd	Positive	HILIC-HRMS	0.15	0.14	0.14	5.57
CMP	Positive	HILIC-HRMS	9.85	8.06	8.96	9.98
DHAP	Positive	GC-MS/MS	0.04	0.05	0.04	12.25
DHIV	Negative	HILIC-HRMS	10.57	6.61	9.51	23.00
FAD	Positive	HILIC-HRMS	1.26	1.34	1.54	10.35
Fuc	Positive	GC-MS/MS	1.08	5.30	4.58	61.89
F6P	Positive	GC-MS/MS	0.25	0.56	0.57	39.67
Fum	Positive	GC-MS/MS	26.63	36.89	31.38	16.23
GA	Positive	GC-MS/MS	2.60	2.51	3.26	14.73
Glc+Man-Ol	Positive	GC-MS/MS	6.50	17.43	11.50	46.34
G6P	Positive	GC-MS/MS	1.18	1.77	1.54	19.66
Glu	Negative	HILIC-HRMS	2015.15	3096.21	2889.74	21.52
Gln	Positive	HILIC-HRMS	966.80	887.86	748.68	12.73
Glu-Cys	Positive	HILIC-HRMS	4.13	4.24	4.88	9.22
GSSG	Positive	HILIC-HRMS	72.02	99.71	106.02	19.54
GSH	Positive	HILIC-HRMS	286.12	280.94	257.31	5.59
G3P	Positive	GC-MS/MS	19.94	17.65	12.70	22.08
Gly	Positive	GC-MS/MS	61.96	63.54	63.66	1.51
Gnin	Positive	HILIC-HRMS	0.11	0.16	0.15	20.42
Gsin	Positive	HILIC-HRMS	0.18	0.18	0.19	2.90
GMP	Positive	HILIC-HRMS	13.24	12.32	13.18	3.99

His	Positive	HILIC-HRMS	83.99	89.71	87.77	3.34
Hse	Positive	HILIC-HRMS	6.17	6.04	5.73	3.80
Isin	Positive	HILIC-HRMS	0.22	0.21	0.23	4.16
IMP	Positive	HILIC-HRMS	0.96	1.42	0.82	29.39
Iso	Positive	GC-MS/MS	1.03	0.54	0.72	32.38
Igsin	Positive	HILIC-HRMS	0.34	0.33	0.34	1.33
Ile	Positive	HILIC-HRMS	14.77	13.53	15.96	8.23
KYN	Positive	HILIC-HRMS	0.80	0.85	0.84	3.06
Leu	Positive	HILIC-HRMS	9.64	11.38	12.94	14.61
Lys	Positive	GC-MS/MS	140.59	151.64	161.91	7.04
Mali	Positive	GC-MS/MS	78.86	135.71	120.25	26.34
Man	Positive	GC-MS/MS	0.28	0.42	0.90	61.34
M6P	Positive	GC-MS/MS	0.34	0.45	0.42	14.45
Met	Positive	HILIC-HRMS	13.42	17.05	18.22	15.40
MVA	Negative	HILIC-HRMS	4.45	5.53	6.84	21.28
OAS	Positive	HILIC-HRMS	1880.31	2859.47	2669.10	21.02
NAD+	Negative	HILIC-HRMS	28.84	26.71	36.58	16.92
NADH	Positive	HILIC-HRMS	125.03	362.02	278.00	47.12
NADP+	Positive	HILIC-HRMS	3.97	3.40	3.37	9.45
NAA	Positive	HILIC-HRMS	6.31	4.18	6.77	24.06
Orn	Positive	HILIC-HRMS	447.68	316.18	487.19	21.47
Phe	Positive	HILIC-HRMS	6.27	9.38	8.78	20.26
Pro	Positive	HILIC-HRMS	265.79	229.94	239.93	7.54
PsU	Negative	HILIC-HRMS	0.77	0.78	0.87	6.67
R5P	Positive	GC-MS/MS	11.84	20.25	16.40	26.06
SAH	Positive	HILIC-HRMS	2.37	2.15	2.29	4.76
S7P	Positive	GC-MS/MS	0.45	2.00	1.68	59.40
Ser	Positive	HILIC-HRMS	155.64	209.45	183.18	14.72
Sped	Positive	HILIC-HRMS	1.89	1.23	3.77	57.35
Suc	Positive	GC-MS/MS	20.71	72.63	112.07	66.92
Thr	Positive	HILIC-HRMS	100.92	106.77	101.79	3.06
Trp	Positive	HILIC-HRMS	1.10	2.04	1.82	29.75
Tyr	Positive	HILIC-HRMS	3.73	7.22	6.47	31.57
Uri	Negative	HILIC-HRMS	0.77	0.78	0.87	6.67
UMP	Negative	HILIC-HRMS	22.21	22.49	28.20	13.90
Val	Positive	GC-MS/MS	41.20	66.37	60.41	23.48

Table S6. In-house yeast-based standard reference materials interlaboratory comparison from some selected metabolites. Quantitative analysis was carried out using both HILIC-HRMS and GC-MS/MS methods with positive mode conditions. Six replicate measurements were performed using the external calibration strategies with additional fully ^{13}C-labeled internal standards (n = 6).

Compound	Abbre.	HILIC-HRMS		GC-MS/MS	
		**Average (nmol/vial)**	**CV (%)**	**Average (nmol/vial)**	**CV (%)**
alpha-Aminoadipic acid	AAD	21.64	3.78	22.67	9.52
alpha-Ketoglutaric acid	AKG	85.51	9.87	79.91	5.75
Cystathionine	CYS	65.65	2.70	60.71	2.71
Fructose-6-phosphate	F6P	1.04	68.77	0.57	6.66
Fumaric acid	Fum	26.89	4.94	31.38	1.19
Homoserine	Hse	5.73	2.81	4.14	3.78
Lysine	Lys	198.33	2.55	161.91	2.34
Methionine	Met	18.22	2.98	23.13	2.10
Phenylalanine	Phe	8.78	2.78	8.30	2.76
Serine	Ser	183.18	2.20	156.06	5.23
Succinic acid	Suc	108.05	3.32	112.07	1.31
Threonine	Thr	101.79	3.24	96.85	3.52
Tryptophan	Trp	1.82	3.22	1.87	7.24

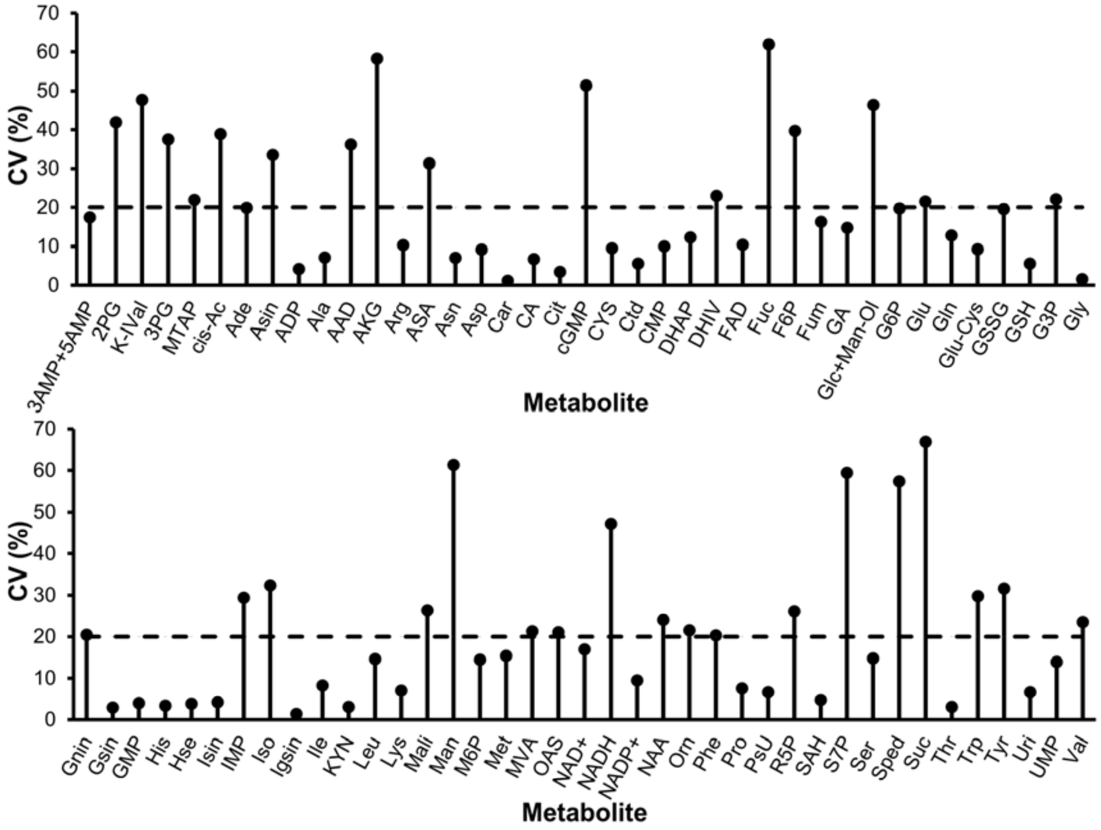

Fig. S1. Inter-batch biological reproducibility for in-house yeast-based standard reference materials. Selected metabolites were quantitatively analyzed by HILIC-HRMS and GC-MS/MS under positive mode conditions. External calibration with the addition of a fully ^{13}C-labeled internal standard was performed for metabolite measurement. The graph shows relative standard deviations (% CV) of metabolites between the different batches (n = 3). The CV calculation is based on the mean values obtained from six vials from each batch. The dashed line indicates a CV of 20%.

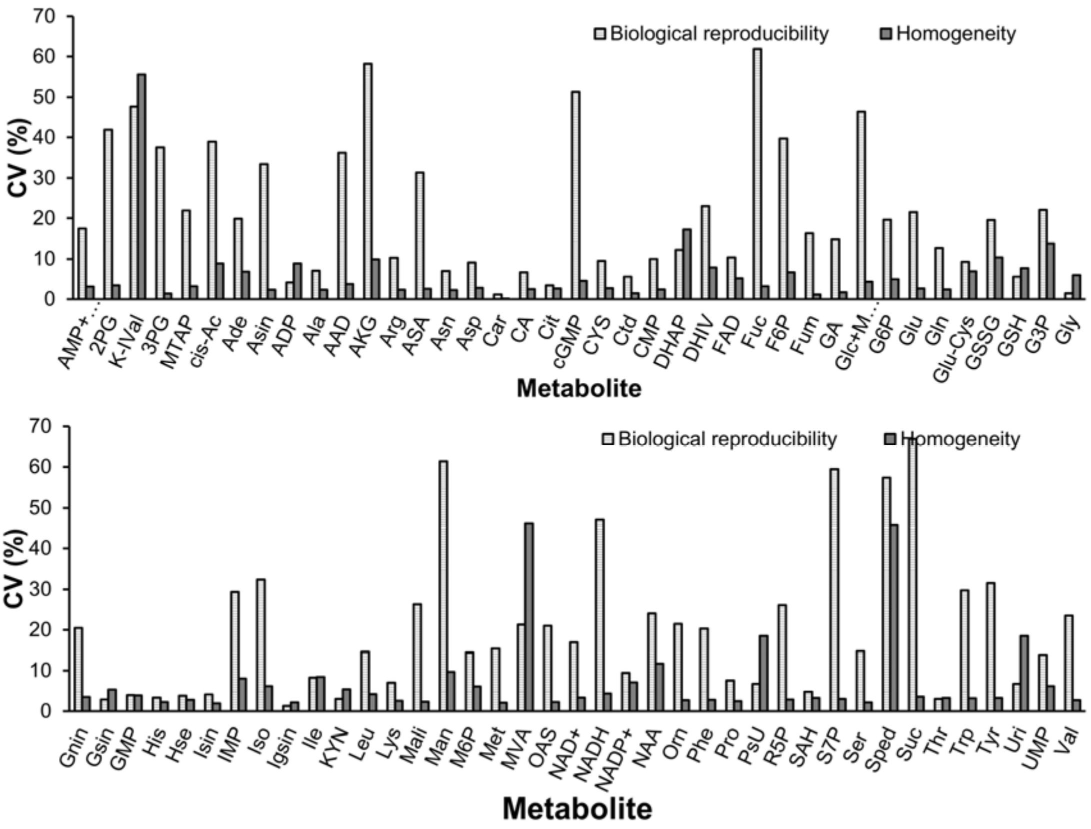

Fig. S2. Variability comparison between biological reproducibility and homogeneity for in-house yeast-based standard reference materials. Metabolite measurement was performed by HILIC-HRMS and GC-MS/MS in positive and negative mode conditions. Six replicate samples with additional fully ^{13}C-labeled internal standards were carried out using the external calibration strategies (n = 6).

Assessment of uncertainty for metabolite quantification in reference material

Uncertainty budget was estimated for threonine as an example of representative metabolite in yeast-based reference material that quantified by GC-MS/MS according to the bottom-up approach in terms of the uncertainty of the measurement and calibration procedure. All possible source parameters associated with concentration quantification (C_{Thr}) using standard calibration were identified, and the standard uncertainty of each input parameter was evaluated, followed by estimated uncertainty contribution (Table 1). Those potential identified input parameters associated with quantity values were the purity of the standard compound (P), the mass of the standard compound (m), the volume of stock standard solution (V_{stock}), and the volume of dilution solvent (V_{dillut}). Other input variables from analytical measurements, such as relative response of the areas in ($RR\ Area_{std}$) in standards and sample ($RR\ Area_{smp}$) contributed to the total combined uncertainty quantification as well.

Table S7. Characterization and quantification of uncertainty source for the preparation of standard calibration for threonine quantification in reference material

Input quantity	Type of distribution	Unit	Value	Absolute standard uncertainty	Relative standard uncertainty (%)	Relative contribution to combined uncertainty[f] (%)
P	Rectangular	%	98.00	1.00[a]	1.02	0.88
m	Rectangular	mg	7.92	0.001[b]	0.01	0.38
V_{stock}	Triangular	µL	162.81	0.60[c]	0.37	0.07
V_{dillut}	Triangular	µL	50.00 - 3711.00	0.20 - 15.00[c]	0.40	0.21
$RR\ Area_{std}$	Normal	-	0.292 - 16.014	0.01 -0.80[d]	3.42-5.00	33.19
$RR\ Area_{smp}$	Normal	-	7.527	0.38[e]	5.05	65.27
**Output quantity[f]**				Total combined	Total combined	
C_{Thr}	-	nmol vial^{-1}	93.54	5.96	6.37	-

[a] Based on the supplier's certificate

[b] Estimated from weighing balance specification

[c] Derived from the pipettes manufacturer specification

[d] Calculated from injected quality control (QC) standard measurement

[e] Observed from the technical replicate sample measurement

[f] Calculated based on propagation procedure using *Monte Carlo* simulation

The established Monte Carlo method was used to estimate total combined measurement uncertainty in this study by randomly generated input quantities within their standard uncertainties. An error propagation procedure with Monte Carlo simulation using @Risk software (Palisade, New York, USA) was performed with 1×10^3 random simulations to estimate variable input quantity that contributed to the total combined uncertainty of metabolite quantification in preparation of standard calibration for metabolite quantification. This simulated method not only provided estimating interaction of all probability distribution functions of input variables involved but also understanding the corresponding uncertainty contributions easily and straightforwardly[1]. In general, the results tabulated in **Table S7** showed that the influence of relative response area from standard calibration and sample that

correspond to the total number of ions measured by the respective instrument were the main sources contributing to the overall combined uncertainty of threonine quantification. Both of them contributed more than 90% of the relative total combined uncertainty for threonine quantification in yeast-based reference material using external standard calibration strategies using a fully ^{13}C-labeled internal standard. The uncertainty related to the weight of the standard metabolite and pipetting volume did not significantly contribute to the total combined uncertainty. Uncertainty calculations associated with the measurement result are systematically assessed by each step of an analytical protocol that contributed to the total uncertainty estimation[2]. In addition, the identification of potential input variables or sources of uncertainty by the bottom-up approach was suitable for improving the experimental procedure with corrective action. This approach also reflected the accuracy of the analytical method used for measurement and comparing determination results from different laboratories[3].

References:

1. Guerrasio R, Haberhauer-Troyer C, Steiger M, Sauer M, Mattanovich D, Koellensperger G, Hann S (2013) Measurement uncertainty of isotopologue fractions in fluxomics determined via mass spectrometry. Anal Bioanal Chem 405:5133–5146 . https://doi.org/10.1007/s00216-013-6910-5
2. Ellison S, Rosslein M, Williams A (2012) EURACHEM/CITAC, quantifying uncertainty in analytical measurement. LGC Teddingt OpenURL
3. Lee S, Choi H, Kim E, Choi H, Chung H, Chung KH (2010) Estimation of the measurement uncertainty by the bottom-up approach for the determination of methamphetamine and amphetamine in urine. J Anal Toxicol 34:222–228 . https://doi.org/10.1093/jat/34.4.222
